# Evaluation of psychological stress in confined environments using salivary, skin, and facial image parameters

**DOI:** 10.1038/s41598-018-26654-4

**Published:** 2018-05-29

**Authors:** Mariko Egawa, Shinichiro Haze, Yoko Gozu, Junichi Hosoi, Tomoko Onodera, Yosuke Tojo, Masako Katsuyama, Yusuke Hara, Chika Katagiri, Natsuhiko Inoue, Satoshi Furukawa, Go Suzuki

**Affiliations:** 1 Shiseido Global Innovation Center, Yokohama, 224-8558 Japan; 2 Japan Aerospace Exploration Agency, Space Biomedical Research Group, Tsukuba, 305-8505 Japan

## Abstract

Detecting the influence of psychological stress is particularly important in prolonged space missions. In this study, we determined potential markers of psychological stress in a confined environment. We examined 23 Japanese subjects staying for 2 weeks in a confined facility at Tsukuba Space Center, measuring salivary, skin, and facial image parameters. Saliva was collected at four points in a single day to detect diurnal variation. Increases in salivary cortisol were detected after waking up on the 4th and 11th days, and at 15:30 on the 1st and in the second half of the stay. Transepidermal water loss (TEWL) and sebum content of the skin were higher compared with outside the facility on the 4th and 1st days respectively. Increased IL-1β in the stripped stratum corneum was observed on the 14th day, and 7 days after leaving. Differences in facial expression symmetry at the time of facial expression changes were observed on 11th and 14th days. Thus, we detected a transition of psychological stress using salivary cortisol profiles and skin physiological parameters. The results also suggested that IL-1β in the stripped stratum corneum and facial expression symmetry are possible novel markers for conveniently detecting psychological stress.

## Introduction

An increasing number of studies have examined health risks to astronauts during space flight as multiple spacefaring nations extend space travel development with a view to undertake human missions to Mars and the Moon. Such missions involve space travel beyond the Earth’s protective magnetosphere into deep space, and studies of human space mission have demonstrated that space exploration involves various health risks^1–3^. The effects of the space environment on human health have mainly been studied in terms of microgravity, cosmic radiation, and closed confined environments. Previous research has reported that microgravity can cause balance disorders, decreased bone mineralisation, and muscle-disuse atrophy^4–8^. In addition, psychological stress in closed, confined, multi-cultural environments in space is a health issue^9–11^. Reasons for the psychological stress in space include isolation from Earth, living and working in a confined environment, low levels of mental and physical stimulation, real danger in space, and in particular, limitation in coping resources to those stressors^9,10^. Until the prolonged space missions, psychological stress was not a serious issue, as astronauts were only able to stay in space for short periods of time. However, the duration of stays on the ISS (International Space Station) has increased in recent years, with some stays nearly a whole year at a time. As longer periods in space will be increasingly required for further space development, such as human explorations to Mars, experiments on the influence of confined environments on the human body have been conducted using various conditions such as SFINCSS-99 (Simulation of Flight of International Crew on Space Station)^12–15^, Mars-500^16–22^, HI-SEAS (Hawaii Space Exploration Analog & Simulation)^23^, Antarctic^24^, and NEEMO (NASA Extreme Environment Mission Operations)^25^. The SFINCSS-99 experiment examined not only group dynamics and group interactions through questionnaires and interviews in an international and confined environment for 240 days, but changes in biomarkers such as plasma volume and urinary catecholamine levels^12–15^. The Mars-500 experiment recorded decreased waking movement, increased crew sedentariness, sleep and rest times by actigraphy^16,17^, increases in plasma^18^/saliva cortisol levels, and heightened immune responses due to prolonged isolation for 520 days^19,22^. An ethological method and wireless group structure module recorded cultural influences and individual differences on crew behavior but did not do so regarding the influence on the frequency of facial expressions and the duration of body interactions^20,21^. The HI-SEAS experiment collected survey evaluations and interviews such as opinions about the modules and exercises^23^. A 12-month stay in Antarctica investigated psychological, social, occupational, and cultural variables^24^.

The Japan Aerospace Exploration Agency (JAXA) has particularly focused research on identifying biomarkers for self-assessment of stress so that astronauts can easily check their stress level by themselves when they are unable to receive regular professional face-to-face interviews on their psychological health by medical doctors. As part of these initiatives, JAXA performs experiments in the confined facility (JAXA-CFE) at the Tsukuba Space Center (Tsukuba, Ibaraki, Japan) that precisely controls the experiment conditions and tracks the subjects’ behaviors in detail. The confined facility designed to simulate a living and working environment in space to examine the influence of psychological stress on the human body^26^. The facility consists of two connected cylindrical rooms, with length, width, and height of 11 meters, 3.8 meters, and 2 meters, respectively. The facility was constructed as a training use mockup of Japanese experimental module “Kibo” in the ISS. The final goal of JAXA-CFE is to develop a handy stress evaluation to be used during orbit that combines low-invasive, time-saving markers with markers that reflect psychological interviews on orbit by expert psychiatrists. The current study was conducted as a part of JAXA-CFE.

The purpose of the current study performed in the JAXA-CFE is to identify potential biomarkers which were not determined or investigated in previous experiments in similar confined environment conditions. We examined diurnal rhythm of salivary cortisol during a 2-week stay in the confined facility by collecting saliva samples four times daily to evaluate influences of confinement. We also examined skin and facial image parameters to explore novel potential markers. We have previously presented preliminary results^27^. We have continued investigations and report the results in this paper.

## Results

### Salivary parameters

**Figure 1:**
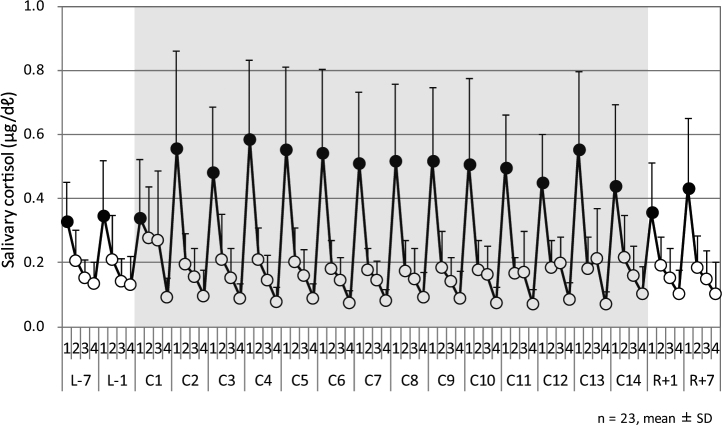
Salivary cortisol profile between 7:00 and 21:00 on each sampling day. L-7 and L-1 were before entering the confined facility, C1–C14 were inside the facility, and R + 1 and R + 7 were after leaving the facility. Sampling time 1 was at 7:00 (60 min after waking up), sampling time 2 was at 12:00 (before lunch), sampling time 3 was at 15:30, and sampling time 4 was at 21:00. Cortisol concentrations are expressed in μg/dℓ.

**Figure 2:**
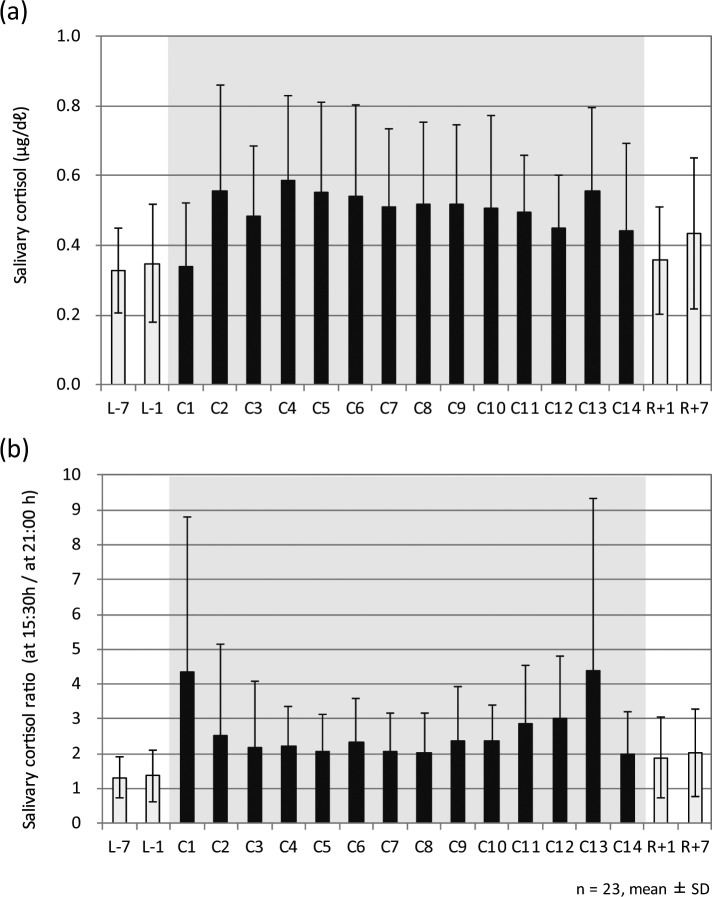
Salivary cortisol concentration at sampling point 1 (at 7:00) (**a**) and ratio of sampling points 3 (at 15:30) and 4 (at 21:00) (**b**).

Figure 1 shows the mean salivary cortisol profile between 7:00 and 21:00 on each sampling day. The mean cortisol profile did not closely follow a circadian rhythm, peaking after awakening and then gradually tapering off over the day. Differences at sampling point 3 (at 15:30) between each sampling day were observed on sampling days C1, C10, C11, C12, and C13. Figure 2(a) represents the mean salivary cortisol concentration at sampling point 1 (at 7:00) on each sampling day. The results indicated that the salivary cortisol surge after awakening increased from C2 to C13, compared with figures obtained outside the confined facility. Table 1(a) represents p-values of the Tukey-Kramer HSD test and Table 2(a) represents pairwise comparisons with the Bonferroni correction between periods in and out of the confined facility of salivary cortisol concentration at sampling point 1 (at 7:00). Tukey-Kramer HSD tests revealed significant differences between sampling day C4 and sampling days L-7 and L-1. In addition, significant differences by pairwise comparisons with the Bonferroni correction were found between sampling days C4 and C11 and sampling day L-7. Figure 2(b) shows the cortisol concentration ratio at sampling points 3 (at 15:30) and 4 (at 21:00). The cortisol concentration ratio on days C1 and C13 were significantly higher according to the Tukey-Kramer HSD test (Table 1(b)) than those obtained outside the confined facility (L-7, L-1, R + 1, and R + 7). Significant differences by pairwise comparisons with the Bonferroni correction (Table 2(b)) between sampling days C6, C10, C11, and C12 and sampling day L-7 were observed. Similarly, significant differences were also observed between sampling days C10, C11, and C12 and sampling day L7.

**Table 1:** P-values from the Tukey-Kramer HSD test.

	C1	C2	C3	C4	C5	C6	C7	C8	C9	C10	C11	C12	C13	C14
**(a)**														
L-7	1.0000	0.0592	0.6515	0.0130*	0.0644	0.1085	0.3493	0.2709	0.2666	0.3645	0.4830	0.9329	0.0617	0.9638
L-1	1.0000	0.1279	0.8366	0.0326*	0.1378	0.2160	0.5518	0.4555	0.4499	0.5694	0.6937	0.9866	0.1327	0.9946
R + 1	1.0000	0.1815	0.9012	0.0508	0.1943	0.2919	0.6561	0.5598	0.5540	0.6730	0.7862	0.9951	0.1878	0.9984
R + 7	0.9940	0.9172	1.0000	0.6555	0.9268	0.9709	0.9995	0.9982	0.9981	0.9996	1.0000	1.0000	0.9220	1.0000
**(b)**														
L-7	0.0001*	0.8834	0.9953	0.9897	0.9991	0.9680	0.9992	0.9995	0.9534	0.9601	0.9820	0.3246	< .0001*	0.9997
L-1	0.0002*	0.9076	0.9973	0.9936	0.9996	0.9776	0.9996	0.9998	0.9663	0.9715	0.9881	0.3571	0.0001*	0.9999
R + 1	0.0063*	0.9999	1.0000	1.0000	1.0000	1.0000	1.0000	1.0000	1.0000	1.0000	1.0000	0.9150	0.0045*	1.0000
R + 7	0.0153*	1.0000	1.0000	1.0000	1.0000	1.0000	1.0000	1.0000	1.0000	1.0000	1.0000	0.9743	0.0111*	1.0000
**(c)**														
L-7								0.9884						0.7577
L-1								0.7654						0.3314
R + 7								0.9997						0.8958
**(d)**														
L-7								0.9801						0.2967
L-1								0.8977						0.1523
R + 7								0.9943						0.8545
**(e)**														
L-7	0.9975			0.2215				1.0000			0.9932			0.9992
L-1	1.0000			0.6071				0.9972			0.8276			1.0000
R + 1	0.7313			0.0159*				0.9964			1.0000			0.7983
R + 7	0.7871			0.0216*				0.9985			1.0000			0.8466
**(f)**														
L-7	0.5890			1.0000				1.0000			1.0000			1.0000
L-1	0.9944			0.9991				0.9980			0.9575			0.9627
R + 1	0.6500			1.0000				1.0000			1.0000			1.0000
R + 7	0.4274			0.9994				0.9998			1.0000			1.0000
**(g)**														
L-7	0.9731			0.9767				1.0000			0.9740			0.9952
L-1	1.0000			1.0000				0.9968			0.6023			1.0000
R + 1	1.0000			1.0000				0.9492			0.3482			0.9999
R + 7	1.0000			1.0000				0.8671			0.2317			0.9986
**(h)**														
L-7	0.8011			1.0000				0.7990			0.9498			0.9999
L-1	0.9664			0.3557				0.9614			0.0095*			0.0359*
R + 1	1.0000			0.9780				1.0000			0.2503			0.6050
R + 7	0.9997			0.9982				0.9997			0.4412			0.8301

Salivary cortisol concentration at sampling point 1 (at 7:00) (a), salivary cortisol concentration ratio of sampling points 3 (at 15:30) and 4 (at 21:00) (b), IL-1β content in the stratum corneum of the forearm (c) and cheek (d), transepidermal water loss (e), sebum content (f), facial expression symmetry parameter A (g), and facial expression symmetry parameter B (h). Asterisk indicates a point of statistical significance (p-value < 0.05).

**Table 2:** Pairwise comparisons with the Bonferroni correction between periods in and out of the confined facility for each parameter.

	C1	C2	C3	C4	C5	C6	C7	C8	C9	C10	C11	C12	C13	C14
**(a)**														
L-7	n.s.	n.s.	n.s.	*	n.s.	n.s.	n.s.	n.s.	n.s.	n.s.	*	n.s.	n.s.	n.s.
L-1	n.s.	n.s.	n.s.	n.s.	n.s.	n.s.	n.s.	n.s.	n.s.	n.s.	n.s.	n.s.	n.s.	n.s.
R + 1	n.s.	n.s.	n.s.	n.s.	n.s.	n.s.	n.s.	n.s.	n.s.	n.s.	n.s.	n.s.	n.s.	n.s.
R + 7	n.s.	n.s.	n.s.	n.s.	n.s.	n.s.	n.s.	n.s.	n.s.	n.s.	n.s.	n.s.	n.s.	n.s.
**(b)**														
L-7	n.s.	n.s.	n.s.	n.s.	n.s.	*	n.s.	n.s.	n.s.	*	*	*	n.s.	n.s.
L-1	n.s.	n.s.	n.s.	n.s.	n.s.	n.s.	n.s.	n.s.	n.s.	*	*	*	n.s.	n.s.
R + 1	n.s.	n.s.	n.s.	n.s.	n.s.	n.s.	n.s.	n.s.	n.s.	n.s.	n.s.	n.s.	n.s.	n.s.
R + 7	n.s.	n.s.	n.s.	n.s.	n.s.	n.s.	n.s.	n.s.	n.s.	n.s.	n.s.	n.s.	n.s.	n.s.
**(c)**														
L-7								n.s.						n.s.
L-1								n.s.						*
R + 7								n.s.						*
**(d)**														
L-7								n.s.						*
L-1								n.s.						*
R + 7								n.s.						n.s.
**(e)**														
L-7	n.s.			n.s.				n.s.			n.s.			n.s.
L-1	n.s.			n.s.				n.s.			n.s.			n.s.
R + 1	n.s.			n.s.				n.s.			n.s.			n.s.
R + 7	n.s.			n.s.				n.s.			n.s.			n.s.
**(f)**														
L-7	*			n.s.				n.s.			n.s.			n.s.
L-1	n.s.			n.s.				n.s.			n.s.			n.s.
R + 1	*			n.s.				n.s.			n.s.			n.s.
R + 7	*			n.s.				n.s.			n.s.			n.s.
**(g)**														
L-7	n.s.			n.s.				n.s.			n.s.			n.s.
L-1	n.s.			n.s.				n.s.			n.s.			n.s.
R + 1	n.s.			n.s.				n.s.			*			n.s.
R + 7	n.s.			n.s.				n.s.			*			n.s.
**(h)**														
L-7	n.s.			n.s.				n.s.			n.s.			n.s.
L-1	n.s.			n.s.				n.s.			n.s.			n.s.
R + 1	n.s.			n.s.				n.s.			n.s.			n.s.
R + 7	n.s.			n.s.				n.s.			n.s.			n.s.

Salivary cortisol concentration at sampling point 1 (at 7:00) (a), salivary cortisol concentration ratio of sampling points 3 (at 15:30) and 4 (at 21:00) (b), IL-1β content in the stratum corneum of the forearm (c) and cheek (d), transepidermal water loss (e), sebum content (f), facial expression symmetry parameter A (g), and facial expression symmetry parameter B (h). Asterisk indicates a point of statistical significance (p-value < 0.05). N.s. is defined as not statistically significant.

### Skin parameters

**Figure 3:**
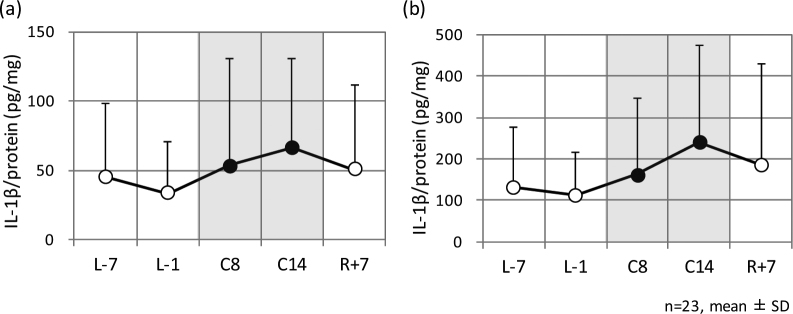
IL-1β content in the stratum corneum on forearm (**a**) and cheek (**b**) on each sampling day. L-7 and L-1 are 7 days and 1 day before entering the confined facility, C8 and C14 are inside the facility, and R + 7 is 7 days after leaving the facility.

Figure 3 shows the mean amount of interleukin-1β (IL-1β) per protein on forearm (a) and cheek (b), respectively, on each sampling day. Increases in the amounts of IL-1β at both sites were observed in the confined environment, and the increase continued until 1 week later. The IL-1β in both the forearm and cheek on C14 were higher than those obtained before/after entering the confined facility. Significant differences in the amount of IL-1β in the forearm between sampling day C14 and sampling days L-1 and R + 7, and those in the cheek between sampling day C14 and sampling days L-7 and L-1 were observed by pairwise comparisons with the Bonferroni correction (Table 2(c,d)). Significant differences were not observed through the Tukey-Kramer HSD test (Table 1(c,d)).

**Figure 4:**
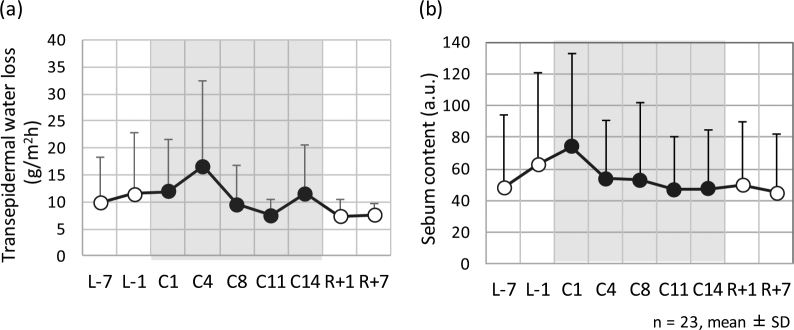
Transepidermal water loss (TEWL) (**a**) and sebum content values (**b**) of the skin on each sampling day. L-7 and L-1 were 7 days and 1 day before entering the confined facility, C1–C14 were inside the facility, and R + 1, R + 7 were 1 day and 7 days after leaving the facility.

Figure 4(a,b) represent the mean transepidermal water loss (TEWL) and sebum content values respectively on each sampling day. TEWL on C4 and sebum content on C1 were significantly higher than those obtained before/after entering the confined facility. Significant differences in TEWL between sampling day C4 and sampling days R + 1 and R + 7 were observed through the Tukey-Kramer HSD test (Table 1(e)). Significant differences in sebum content were observed between sampling day C1 and sampling days L-7, R + 1, and R + 7 by pairwise comparisons with the Bonferroni correction (Table 2(e)).

### Facial expression symmetry

**Figure 5:**
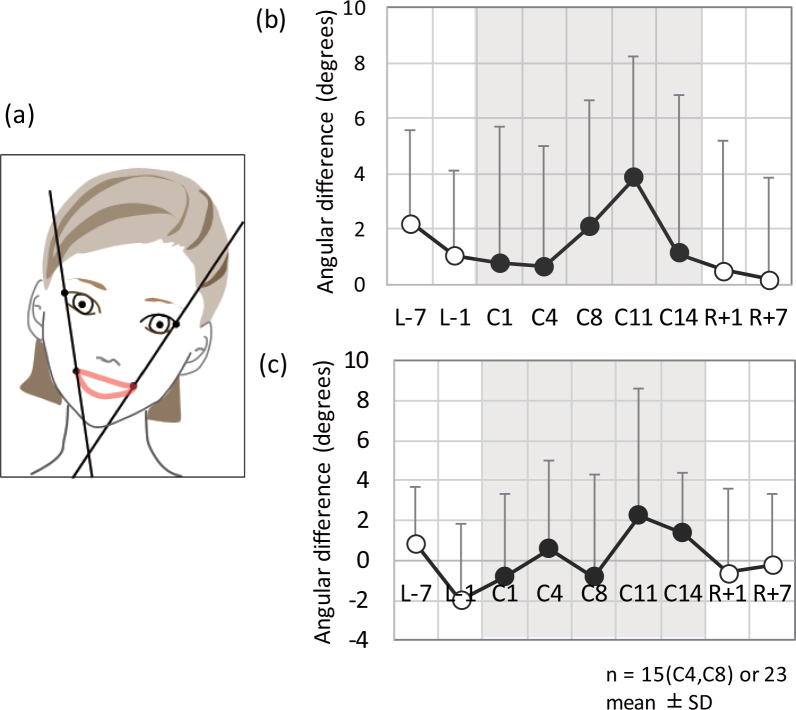
Facial expression symmetry parameters on each sampling day: overview of each parameter (**a**), parameter A (**b**), and parameter B (**c**). Parameter A represents differences in the left and right angles of the lines connecting the corner of the mouth with the corner of the eye from the horizontal and parameter B, differences in parameter A between a serious expression and a smiling expression. L-7 and L-1 are 7 days and 1 day before entering the confined facility, C1–C14 are inside the facility, and R + 1 and R + 7 are 1 day and 7 days after leaving the facility.

Figure 5 shows the facial expression symmetry parameters on each sampling day. An overview of each parameter is shown in Fig. 5(a). Parameter A represents the left and right differences in the angles of the line connecting the corner of the mouth with the corner of the eye from horizontal. Parameter B represents differences in parameter A between serious and smiling expressions. Figure 5(b) shows parameter A and Fig. 5(c) shows parameter B. The facial expression symmetry parameters in C11 and C14 were significantly higher than those obtained outside of the confined facility. Significant differences were observed in parameter A between sampling day C11 and sampling days R + 1 and R + 7 by pairwise comparisons with the Bonferroni correction (Table 2(g), and in parameter B between sampling days C11 and C14 and sampling day L-1 by the Tukey-Kramer HSD test (Table 1(h)).

## Discussion

In the current study, we found possible non-invasive biomarkers to measure the effects of psychological stress in the confined environment. Psychological stress is also a major health issue in daily life because of its involvement in multiple physiological and psychological illnesses, particularly in industrialized societies^28–30^. Saliva is one of the most easily collected sample types, and various proteins in saliva have been found to change with stress in daily life conditions. Cortisol, α-amylase, chromogranin A, and immunoglobulin A have all been identified as salivary stress markers^31^, and salivary cortisol is a widely used measure^30,32,35^. In the current study, we used salivary cortisol as a possible stress marker because salivary cortisol has been widely used as a marker in stress research in daily life conditions^36–38^. Salivary cortisol has been found to increase with acute stress^30^. In contrast, chronic stress has mainly been evaluated under real-life stressful conditions using the flattened diurnal curve of cortisol release and the area under the curve of cortisol release within a day^30,32–40^. Cortisol release typically follows a circadian rhythm, peaking at 30–60 min after awakening then gradually tapering off over the day. We observed an increase in cortisol surge in the morning, which may have been related to leading a regular life style pattern in confined environments or to the influence of stress. A previous study of measurement in a confined environment on plasma/saliva cortisol in the morning also showed an increase in cortisol in the morning^18,22^. Furthermore, a previous study of diurnal variation in cortisol release among medical graduates reported an increase in cortisol levels in the afternoon^30^. The researchers speculated that stressful exams immediately before the sampling of saliva may have influenced cortisol increase. The current results revealed an increase in cortisol content at sampling point 3 (at 15:30) on day C1, which was the day participants entered the confined facility. The significant increase in the cortisol value at sampling point 3 (at 15:30) was also observed in the second half of the stay. Changes in diurnal variation in cortisol release were observed not only immediately after entering the confined facility, but also in the latter half of the stay in the facility, which may reflect a stronger intensity of stress in the second half. The increase disappeared on day C14, which was the day participants left the facility. Psychological factors regarding the end of the experiment might have an effect on cortisol responses on day C14. Considering that the diurnal variation in cortisol are affected by the psychological conditions of the subjects, it would be a sensitive marker to detect psychological stress.

However, it would be much better if we could use markers which do not require sampling multiple times in a day. Therefore, we tried to find other non-invasive markers. Several studies have examined the effects of psychological stress on the skin, indicating that stress exacerbates several skin disorders. Interview stress caused delayed recovery of the barrier function of the skin, increased plasma cortisol levels, and activation of several inflammation and immune systems, including IL-1β and IL-10, in the blood^28^. The mechanisms contributing to acute psychological stress-induced exacerbation of inflammatory skin disorders, including psoriasis, eczema, atopic dermatitis, and aggravated contact dermatitis, have been suggested to be stress-induced impairments of skin permeability barrier homeostasis^28,39,40^. Even for healthy subjects, it was reported that final examination stress on students^41^, or marital difficulty^42^, delayed skin barrier recovery. In the current study, we observed significant changes in biomarkers related to the skin barrier function, such as increases in IL-1β in the stratum corneum on day C14 and in TEWL values which indicated degradation of the skin barrier function on day C4. IL-1β influences the barrier function through an inflammatory reaction, and TEWL represents the barrier function of the skin. In addition, the amount of dead stratum corneum may be influenced by the turnover of the skin. Therefore, the timing of the increase of these two markers would be expected to differ. A pilot study of skin physiological parameters, including TEWL, examined the influence on astronauts of staying on the ISS for a maximum of 159 days^43^. The results showed that mean TEWL values post-flight were significantly higher than pre-flight values. Although the experimental conditions were different from those in the current study, we observed a similar trend toward changes in TEWL values. Thus, the biomarkers related to the skin barrier function would be helpful to measure the effects of psychological stress. Regarding sebum, we observed an increase on day C1. In a study of stress among students, increased acne severity was associated with stress levels^44^. Another study reported an association between psychological stress and the severity of acne, especially in males, but several factors other than sebum quantity were also suggested to influence acne severity^45^. Thus, the mechanisms underlying the acute change we observed in sebum content on C1 are currently unclear. One possibility is the involvement of adrenergic androgen, which stimulates sebaceous gland activity^46^.

Finally, we determined the possibility of using facial image parameters to detect the effects of psychological stress, which could potentially provide remote, convenient tools for evaluating stress levels without cumbersome biochemical analysis. Facial expression is often used to evaluate pain in non-communicative critically ill patients in clinical settings, and a previous study reported that upper facial expressions were most frequently activated during pain responses^47^. Facial expression-related parameters have also been studied in the field of psychology and have been used as indices to represent various brain states^48,49^. Potential associations have been reported between the direction of anatomical asymmetries of the facial skeleton and frontal lobe at the individual level in a study using gorillas^48^. Another study in humans reported left hemi-face dominance, by measuring facial electromyographic asymmetry during corrugator activity in high and low arousal negative emotion blocks^49^. In addition, it has been reported that healthy older adults exhibit increased responsivity of brain regions involved in face and emotion processing while under stress^50^. Based on these findings, in the current study, we focused on the effects of psychological stress on facial expression symmetry while subjects were smiling, which would be expected to stress the movement of muscles. Parameter A included the influence of behaviour patterns when subjects faced the screen directly in addition to facial expression symmetry while smiling. As parameter B indicated the difference between smiling and a serious expression, it only extracted facial expression symmetry during facial expression change. The current study clarified that both parameters changed in the confined environment. Facial images can be easily acquired during space missions. Further possibilities to apply the markers to stress conditions from daily life would also be worth considering.

Regarding differences in the p-value between the Tukey-Kramer HSD and pairwise comparisons with the Bonferroni correction, they would relate to variations in the eigenvalues of individuals in addition to the detection sensitivity of each statistic. For example, facial image parameter A contains original facial distortion of the subject which includes individual differences, so pairwise comparisons with the Bonferroni correction are suitable for this type of marker. Using only significant statistical differences to select markers would not be a sufficient way to judge each stress marker. P-values by Tukey-Kramer HSD were also shown in the results to be an easy point of comparison regarding the sensitivity of the markers. In future studies, we should also consider the effect of differences in behavior patterns by nationality, diverse or homogenous groups, gender, and prolonged stay in a confined environment.

In conclusion, we found possible biomarkers to measure psychological stress in confined environments using salivary cortisol profiles, IL-1β in the stripped stratum corneum, and the skin’s physiological parameters. We also examined potential novel markers using facial expression symmetry as more convenient measures. Combining multiple markers may also be useful for monitoring detailed changes in various states of psychological stress in confined environments. However, further detailed analysis is necessary to confirm this through comparisons to other markers such as other physiological markers, psychometric scales, and interviews by psychiatrists.

## Methods

### Experimental procedures

JAXA-CFE was started from February 2016. Eight subjects per experiment stayed together in the confined facility (JAXA) for 2 weeks (days: C1–C14). The confined facility contained several monitoring cameras and microphones. We provided instructions to the subjects from the control room through speakerphones or interphones. A video conferencing system enabled two-way video interviews between the confined facility and the control room. The daily schedule for subjects was controlled, including meals, waking up time (at 6:00), bedtime (at 22:00), and daytime tasks. The procedure follows that described in a previous publication^23^. Physiological markers such as blood, urine, and saliva, psychometric scales, and interviews by psychiatrists were obtained by participating research teams. In the current study, the experiment conducted in February and September 2016, and February 2017 were analysed. As one subject in the test conducted in September 2016 withdrew the study due to acute enteritis on day C2, twenty-three healthy Japanese subjects (19 men and four women; age range: 20–55 years; mean age: 37.4 years) participated in the confined environment stress study. Measurements were performed 7 days and 1 day before (L-7, L-1) and 1 day and 7 days after (R + 1, R + 7) entering the confined facility to obtain baseline data in addition to inside the facility (days: C1–C14). All measurements were performed in an air-conditioned room at approximately 22–24 °C and 24–43% RH. The test protocol was performed in accordance with the principles of Declaration of Helsinki and approved by the Ethics Committee of JAXA and SHISEIDO Co. Ltd. Subjects were informed the purpose of the study, and written informed consent was obtained prior to the participation.

### Cortisol in saliva

Saliva was collected in a tube at the following four points on all sampling days using the passive drool method: (1) at 7:00 (60 min after waking up); (2) at 12:00 (before lunch); (3) at 15:30; and (4) 21:00. Each sample served as its own control. The collected saliva was stored at −80 °C until further analysis. A salivary cortisol enzyme-linked immunosorbent assay (ELISA) kit (expanded range high sensitivity, Salimetrics LLC, State College, PA) was used for evaluation of salivary cortisol content.

### IL-1β in the stratum corneum

Stripped stratum corneum was obtained by pressing the adhesive surface of scotch tape (Nichiban Co. Ltd., Tokyo, Japan) to the skin surface on the left side of the face and inner side of the left forearm^51^. The sampling was performed immediately after the skin measurements on days L-7, L-1, C8, C14, and R + 7 in the morning. The tape was firmly attached to the skin and repeatedly pressed with fingers over the entire area. After sampling, pieces of scotch tape were stuck on plastic sheets and stored at −80 °C. Human IL-1β/IL-1F2 QuantiGlo ELISA Kit (R&D Systems, Inc., Minneapolis, MN) was used for evaluation of IL-1β in the stratum corneum. After removal of the tape from the plastic sheet, a 14.4 cm^2^ (2.4 cm × 6 cm) section of tape was cut into small pieces and immersed in 1 ml of extraction buffer (0.1 M Tris–HCl, pH 8.0 + 0.14 M NaCl + 0.1% Tween 20). The samples were sonicated four times for 30 sec each, and an extract of the stratum corneum was obtained via centrifugation. Protein concentration was measured using a DC protein assay kit (Bio-Rad Laboratories, Inc., Hercules, CA) according to the manufacturer’s instructions. The IL-1β amount per total protein amount was used as the IL-1β concentration.

### Skin physiology

Skin parameters reflecting skin physiology were non-invasively measured on days L-7, L-1, C1, C4, C8, C11, C14, R + 1, and R + 7. The measurements were performed just after the sampling of saliva at time point (1) (at 7:00). TEWL was measured on the left volar forearm to evaluate skin barrier function using a VapoMeter^®^ (Delfin Technologies Ltd., Kuopio, Finland). Skin surface sebum content was measured on the left side of cheek using Sebumeter^®^ (Courage + Khazaka Electronic GmbH, Cologne, Germany). Topical application of medicine and cosmetics to the measurement area after washing the face in the morning or taking a shower in the previous night was prohibited until the measurement finished.

### Facial expression symmetry

Facial expression symmetry at the time of facial expression change was evaluated on days L-7, L-1, C1, C4, C8, C11, C14, R + 1, and R + 7. A laptop computer (VAIO, Sony Corporation, Tokyo, Japan), a web camera (LifeCam Studio, Microsoft Corporation, Redmond, WA), and specialised image acquisition software (Koozyt Inc, Tokyo, Japan) were used to obtain facial images. The subjects were instructed to adjust their faces horizontally in the centre of the display windows. A black mask was added at the centre of the facial images so that subjects could not see their expression images. Subjects were instructed to produce two facial expressions: a “serious expression” and a “smiling expression (the biggest smile you can make)” six times, maintaining the expression for 3 sec each time. The recording was performed during free time, while subjects were in a relaxed condition. Left and right distortion of facial expression was analysed based on the angular difference between the corner of the eye and mouth using facial images collected during the 2nd and 3rd trials, and averaged.

### Statistical Analysis

IBM® SPSS® Statistics (ver.23.0.0.0, IBM Corp., NY) and JMP® (ver.13.2.1, SAS Institute Inc., NC) were used for statistical analysis. The Tukey-Kramer test was used for multiple comparisons of group means. Multiple pairwise comparisons using Bonferroni corrections were conducted to determine changes in confined environments to the baseline. A value of P < 0.05 was considered statistically significant.
